# Acute Pleuropericardial Syndrome Following Messenger Ribonucleic Acid (mRNA) COVID-19 Vaccination: A Case Report

**DOI:** 10.7759/cureus.88611

**Published:** 2025-07-23

**Authors:** Odunayo Yusuf, Ajibola Omotosho, Sarah Moore, Aisha Khan

**Affiliations:** 1 Internal Medicine, Manchester University NHS Foundation Trust, Manchester, GBR; 2 Internal Medicine, North Manchester General Hospital, Manchester, GBR; 3 Rheumatology and General Internal Medicine, North Manchester General Hospital, Manchester, GBR; 4 Acute Internal Medicine, Manchester University NHS Foundation Trust, Manchester, GBR

**Keywords:** covid-19 vaccine complication, pericardial diseases, pericardial tamponade, pleuropericardial effusion, rare cause of pleural effusion

## Abstract

Pleuropericardial syndrome, a rare inflammatory complication associated with messenger ribonucleic acid (mRNA) COVID-19 vaccination, is predominantly reported in younger men but may also occur in older adults following booster doses, as illustrated by this case of a 68-year-old woman. Three days after receiving her third (booster) dose, she presented with flu-like symptoms, palpitations, and progressively worsening dyspnea. Diagnostic imaging revealed a large pericardial effusion and a moderate pleural effusion. Emergent pericardiocentesis was performed, draining 850 mL of serosanguinous fluid. Subsequent anti-inflammatory therapy led to complete resolution of symptoms, with no recurrence over a 15-month follow-up period.

This presentation is likely driven by immune-mediated serosal inflammation, potentially triggered by the activation of innate immune pathways or molecular mimicry following mRNA vaccination. This case underscores the importance of prompt recognition and thorough differential diagnosis, even in atypical patient demographics such as older women postbooster vaccination. It further highlights the need for systematic reporting of such adverse events to pharmacovigilance systems, thereby supporting ongoing vaccine safety monitoring and informing future booster strategies.

## Introduction

Myopericarditis, which includes both myocarditis and pericarditis, has been recognized as a rare but important adverse event following messenger ribonucleic acid (mRNA) COVID-19 vaccination. Surveillance data from systems such as the Vaccine Adverse Event Reporting System (VAERS) in the United States and the Medicines and Healthcare products Regulatory Agency (MHRA) Yellow Card scheme in the United Kingdom estimate the incidence of myocarditis and pericarditis to range from approximately 12.6 cases per million doses in young men to substantially lower rates in older adults [1,2]. However, pleuropericardial syndrome, involving simultaneous inflammation and effusions of the pericardial and pleural cavities, remains exceptionally rare, with limited epidemiological data available.

Most reports of vaccine-associated serosal inflammation involve younger men after the second vaccine dose [3]. Presentations in older adults, women, and particularly following booster doses, are much less well described. The current case of a 68-year-old woman who developed acute pleuropericardial syndrome three days after receiving an mRNA COVID-19 booster dose represents a less commonly documented patient demographic and timing, providing important clinical insight into the broader spectrum of vaccine-related inflammatory responses.

Recent case series and surveillance data suggest that adverse events following booster doses are largely consistent with those after the primary vaccination series, although rare inflammatory conditions, such as polyserositis, have been infrequently reported [4]. This highlights the importance of continued monitoring and detailed characterization of such cases.

To ensure the accurate detection and effective management of these rare complications, systematic reporting through formal pharmacovigilance frameworks, such as VAERS, the MHRA Yellow Card scheme, and other international registries, is essential. These systems enable robust postmarketing safety surveillance, helping to define the true incidence, identify populations at risk, and inform future vaccine policies.

## Case presentation

A 68-year-old woman with no significant past medical history presented to the emergency department with a two-day history of flu-like symptoms, intermittent palpitations, and dyspnea. These symptoms began three days after she received a third (booster) dose of an mRNA COVID-19 vaccine. Initial clinical assessment and electrocardiogram confirmed paroxysmal atrial fibrillation without signs of heart failure. She was discharged on bisoprolol and edoxaban, with an outpatient transthoracic echocardiogram (TTE) arranged.

Over the following week, her dyspnea progressively worsened, prompting private cardiology review, during which a cardiac magnetic resonance imaging revealed a large pericardial effusion, and she was immediately admitted for further evaluation. On examination, she was afebrile but dyspneic at rest with elevated jugular venous pressure visible to the angle of the jaw and distended neck veins. Heart sounds were muffled, and breath sounds were absent at the left lung base. Her blood pressure remained stable, and there was no peripheral edema.

**Figure 1:**
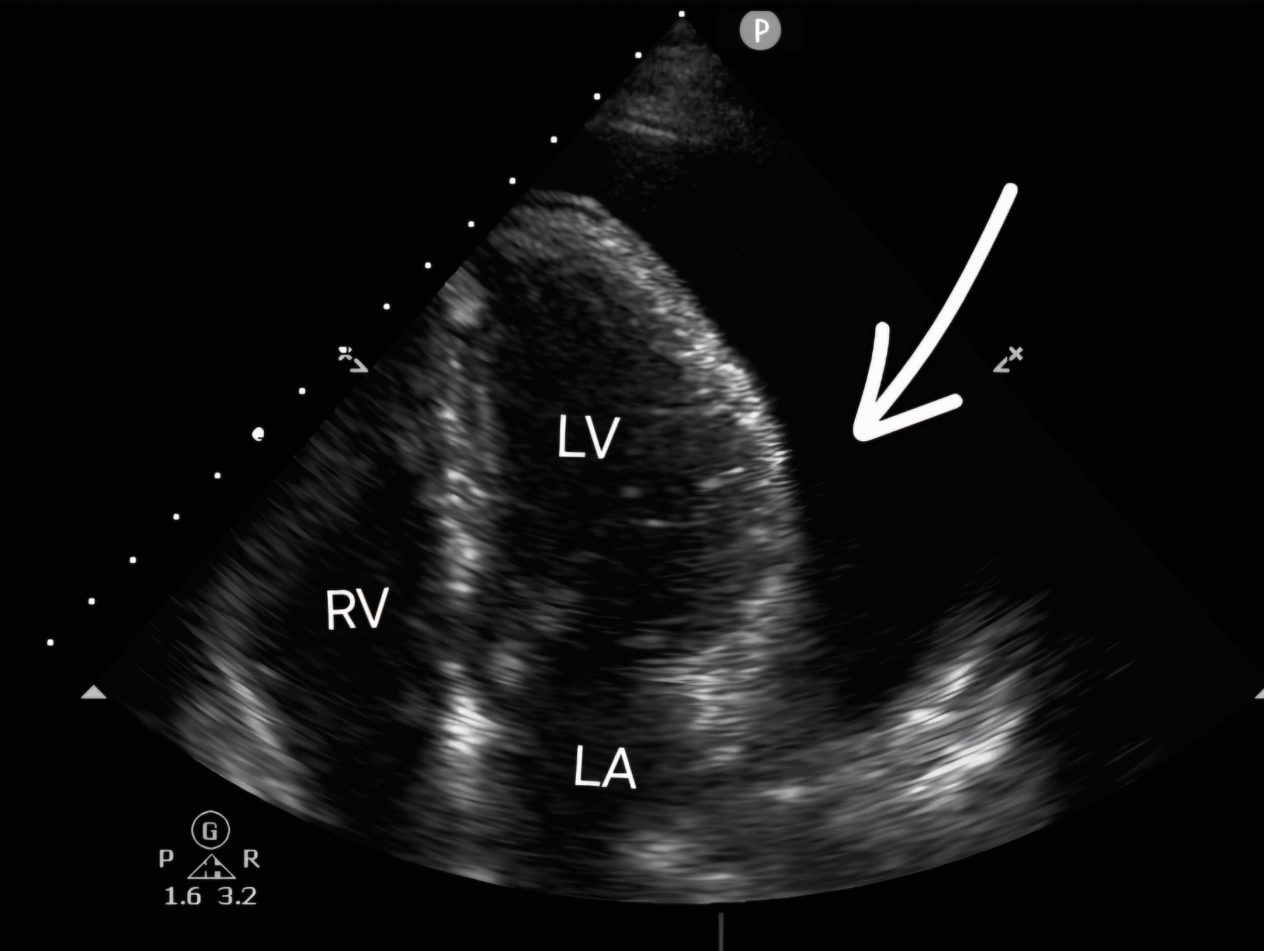
Apical four-chamber transthoracic echocardiogram showing a large circumferential pericardial effusion (white arrow), visible as an anechoic space surrounding the heart. The RV appears compressed, consistent with diastolic collapse, a hallmark of cardiac tamponade. The LV and LA remain structurally preserved RV: right ventricle; LV: left ventricle; LA: left atrium

Bedside transthoracic echocardiography confirmed a large pericardial effusion alongside a moderate left pleural effusion, with no initial echocardiographic signs of tamponade. The patient was admitted for cardiac monitoring, pericardiocentesis, and further investigations. At this stage, her anticoagulant therapy was discontinued. By the following morning, she developed tachycardia and a progressive decline in blood pressure, prompting a repeat TTE (Figure 1). This showed an increased pericardial fluid volume with new features consistent with cardiac tamponade, while the left pleural effusion remained moderate in size.

**Figure 2:**
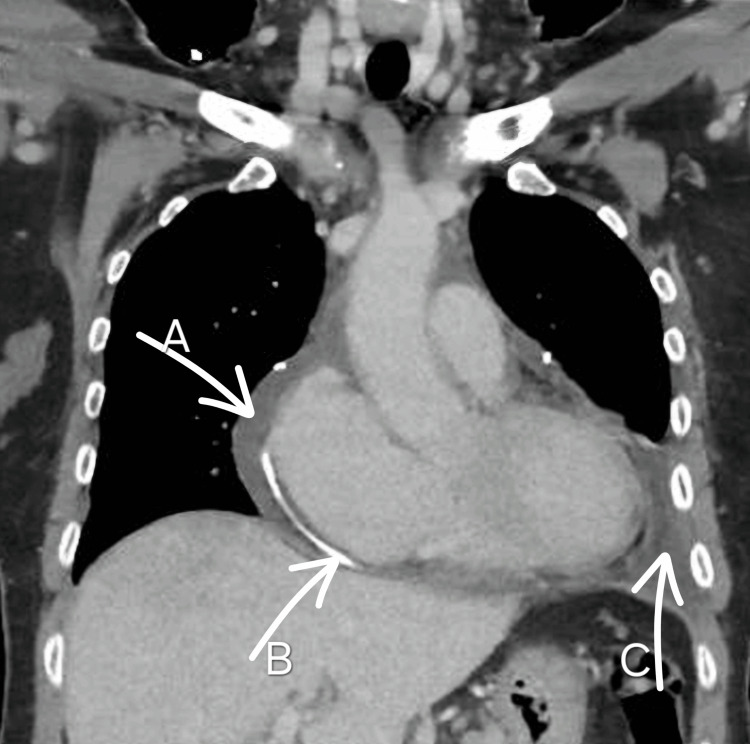
Contrast-enhanced coronal CT image of the thorax, showing a moderate pericardial effusion (arrow A) and a left-sided pleural effusion (arrow C). Notably, a pigtail catheter (arrow B) is visualized within the pericardial space, indicative of ongoing or recent pericardiocentesis for therapeutic drainage CT: computed tomography

Urgent pericardiocentesis was performed under echocardiographic guidance and immediately yielded 450 mL of blood-stained fluid. A pigtail catheter was left in situ for ongoing drainage. By the third day of hospital admission, a total of 850 mL of serosanguinous fluid had been drained, and the catheter was removed. Pleural aspiration was deferred given the moderate volume and the patient’s mild respiratory symptoms. This was supported by thoracic computed tomography (CT) imaging (Figure 2), which confirmed the effusion size.

Pleural aspiration was not performed during the patient’s hospitalization. A pleural consultant reviewed the case and concluded that the moderate pleural effusion, in the context of acute serositis affecting both the pericardium and pleura (pleuropericardial syndrome), was likely inflammatory and self-limiting. Given the availability of diagnostic pericardial fluid analysis, the moderate size of the pleural effusion, and the absence of significant respiratory symptoms, pleural aspiration was deemed unlikely to provide additional diagnostic benefit. This approach aligns with clinical experience that pleural fluid cytology in such cases typically mirrors pericardial fluid findings. We acknowledge that this limits the full characterization of the pleural component.

Biochemical analysis of the pericardial fluid (Table 1) fulfilled Light’s criteria for an exudate [5]. The earlier CT of the thorax, abdomen, and pelvis also demonstrated mediastinal and thoracic lymphadenopathy with benign reactive features and no evidence of malignancy. Cytological examination of the pericardial fluid revealed a predominance of CD68-positive macrophages. Immunohistochemistry was negative for calretinin, thyroid transcription factor-1, BerEP4, carcinoembryonic antigen, and GATA binding protein 3, findings consistent with a benign inflammatory process.

**Table 1:** Biochemical analysis of serum and pericardial fluid in the evaluation of pericardial effusion Serum and pericardial fluid biochemical parameters measured to assess the nature of pericardial effusion. Elevated serum and pericardial fluid LDH levels, along with the LDH ratio >0.6 and protein ratio >0.5, support the diagnosis of an exudative effusion. Reference ranges are provided for comparison where applicable LDH: lactate dehydrogenase

Investigation	Result	Reference range and units
Serum LDH	2,445 U/L	125-250 U/L
Pericardial fluid LDH	2,032 U/L	-
Pericardial fluid LDH to serum LDH ratio	0.8	>0.6 for exudative effusion
Serum total protein	73 g/dL	60-80 g/dL
Pericardial fluid total protein	52	-
Pericardial fluid total protein to serum total protein ratio	0.7	>0.5 for exudative effusion

Comprehensive infectious screening included an atypical pneumonia panel, pericardial fluid cultures including mycobacterial cultures, QuantiFERON-TB Gold, HIV-1 and HIV-2 serology, a viral hepatitis panel and extended respiratory viral polymerase chain reaction (PCR) covering SARS-CoV-2, influenza A and B, respiratory syncytial virus, rhinovirus and enterovirus, parainfluenza virus, adenovirus, human metapneumovirus, Epstein-Barr virus, and cytomegalovirus. All results were negative.

Autoimmune serology, including antinuclear antibodies, extractable nuclear antigen profile, myeloperoxidase, proteinase 3, rheumatoid factor, centromere antibodies, and complement levels (C3 and C4), was within normal limits. Serum biochemistry revealed no metabolic derangements, with normal thyroid function tests and glycated hemoglobin levels. Notably, C-reactive protein was elevated at 33 mg/L, while erythrocyte sedimentation rate was 16 mm/hour. Serum troponin and brain natriuretic peptide levels and other biochemical results were within normal range.

The pleural medicine team diagnosed acute serositis (pleuropericardial syndrome) of inflammatory etiology and prescribed a 10-day course of oral nonsteroidal anti-inflammatory drugs (NSAIDs). While NSAID use in this context is largely based on extrapolation from pericarditis management rather than direct evidence, they were chosen to address inflammation and symptoms. Given the clear temporal association with mRNA vaccination, the presentation was considered vaccine-related, and an adverse event report was submitted to the MHRA via the Yellow Card scheme.

**Figure 3:**
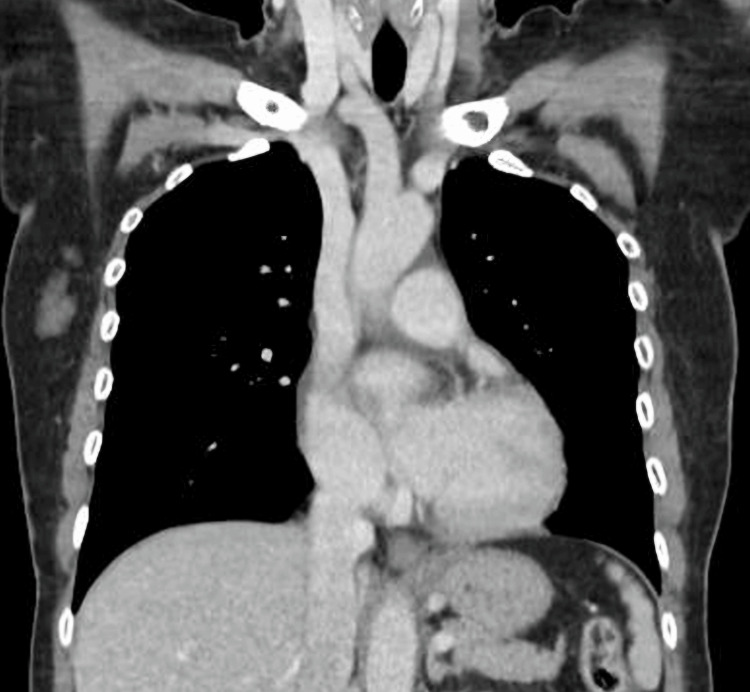
Coronal CT thorax demonstrating complete resolution of pleural and pericardial effusions The effusions are similar to those observed in Figure 2 CT: computed tomography

Her symptoms resolved completely within two weeks. Over 15 months of follow-up, serial echocardiography and chest CT scans (Figure 3) demonstrated no recurrence of pericardial or pleural effusions, and mediastinal lymphadenopathy resolved. She was subsequently discharged from follow-up with no further complications.

## Discussion

Involvement of both the pericardial and pleural spaces by inflammatory fluid is uncommon but a clinically important manifestation of serosal inflammation. Such dual compartment effusions are most often seen in systemic conditions, including autoimmune disorders, infections, malignancy, or after cardiac injury. While individual pericardial or pleural effusions are relatively common, their concurrence should prompt comprehensive evaluation to exclude tuberculosis, malignancy, and connective tissue disease [5,6]. Although most reports of postvaccination serosal inflammation involve young men after primary doses, this case of an older woman presenting three days following a booster dose highlights the need for vigilance across all demographics. The transient thoracic lymphadenopathy and predominance of CD68-positive macrophages in pericardial fluid further support a macrophage-driven inflammatory response, even though the absence of pleural fluid sampling limits full characterization.

SARS-CoV-2 infection itself has been linked to serosal inflammation, with pericardial effusion occurring in approximately 4%-5% of hospitalized patients and occasional pleural involvement [7]. With the introduction of mRNA vaccines, rare cases of myocarditis and pericarditis have been described, particularly in young men after second doses [1,2]. Proposed mechanisms include immune activation by lipid nanoparticle delivery systems, persistence of spike protein antigens, and molecular mimicry, but these remain speculative and may not fully explain isolated pleuropericardial presentations in older adults [8-10].

Several case reports have described pleuropericardial effusions following mRNA COVID-19 vaccination. Oudah et al. detailed a 78-year-old man who developed unilateral pleural effusion without identifiable infection or malignancy [11], and Mizoguchi et al. reported bilateral pleural and pericardial effusions in an 88-year-old woman [12]. All resolved with anti-inflammatory therapy and supportive care, supporting an inflammatory rather than infectious or neoplastic etiology [11,12].

The differential diagnosis for pleuropericardial effusion is broad. Viral pericarditis due to pathogens such as coxsackievirus, echovirus, and influenza remains common, and tuberculosis must be excluded, especially in endemic areas, using staining, culture, and PCR [5,6]. Autoimmune serositis, particularly from systemic lupus erythematosus, and neoplastic causes from lung, breast, or lymphoid malignancies should also be considered and excluded with serology, imaging, and cytology [13].

In our patient, comprehensive exclusion of infectious, malignant, and autoimmune causes was achieved. Echocardiography-guided pericardiocentesis yielded serosanguinous fluid that fulfilled exudative criteria, and cytology demonstrated CD68-positive macrophages, with no granulomas or malignant cells. A 10-day course of NSAIDs led to rapid symptom resolution, consistent with prior reports of postvaccine serositis [11,12].

Long-term follow-up in such cases appears favorable, with no documented progression to constrictive pericarditis or fibrothorax. However, the available data derive from small series with limited follow-up, so the absence of chronic sequelae should be interpreted with caution. Prompt recognition and early intervention remain crucial to optimize outcomes.

Adverse events, such as pleuropericardial syndrome, should continue to be reported through established pharmacovigilance systems. The development of structured registries and the aggregation of similar cases will be essential to define the true incidence, elucidate mechanisms across different populations, and guide future booster recommendations and risk communication [14].

Clinicians should maintain a high index of suspicion for pleuropericardial inflammation in patients presenting with dyspnea, pleuritic chest pain, or symptoms of fluid overload after vaccination. Ongoing data collection and targeted mechanistic studies will be key to refining prevention and management strategies while preserving confidence in vaccine safety.

## Conclusions

This case underscores acute pleuropericardial syndrome as an uncommon but important inflammatory complication following (mRNA) COVID-19 booster vaccination. While most reports of postvaccination serosal inflammation involve younger men and are limited to myocarditis or pericarditis, this case illustrates that older adults and women may also develop clinically significant pleural and pericardial involvement. A thorough diagnostic workup is essential to exclude alternative etiologies such as infection, malignancy, or autoimmunity, and echocardiography remains central to both diagnosis and management.

Prompt recognition and early intervention with pericardiocentesis and anti-inflammatory therapy led to full recovery in this patient, with no recurrence over prolonged follow-up. Although causality cannot be definitively established, the close temporal association, exclusion of alternative causes, and benign inflammatory profile meet the World Health Organization Uppsala Monitoring Centre criteria for a “probable” adverse drug reaction. Nonetheless, single case reports cannot infer population-level risk. Enhanced pharmacovigilance, case aggregation, and development of structured reporting systems are warranted to better delineate patterns of postvaccination serosal inflammation and guide evidence-based booster strategies.
